# Vasoplegic Syndrome Post-cardiopulmonary Bypass in a Renal Transplant Patient: The Brain Is Not the Index Organ

**DOI:** 10.7759/cureus.21280

**Published:** 2022-01-15

**Authors:** Sean R Bennett, Julia Gonzalez, Jose A Fernandez

**Affiliations:** 1 Anesthesiology, King Faisal Cardiac Center, King Abdulaziz Medical City, National Guard Health Affairs, Jeddah, SAU

**Keywords:** methylene blue infusion, cardiopulmonary bypass, multiorgan failure, cerebral oximetry, vasoplegic syndrome, autobiographical case report

## Abstract

Vasoplegia syndrome (VS) is seen in cardiac surgery post-cardiopulmonary bypass (CPB) and defined by increasing requirements for more than one vasoactive agent to which the patient's response is reduced. It is also associated with normal or high cardiac output (CO). Prolonged CPB time is the second commonest precipitating factor. Here, we describe a young adult, with good right ventricular (RV) and left ventricular (LV) function, who previously was a renal transplant recipient with a functioning kidney who developed VS and shock after CPB to replace the mitral and aortic valves. During the first two hours of CPB, his mean arterial blood pressure (MAP) was never lower than 50 mmHg. His brain regional cerebral oxygen saturation (rSO_2_) remained above baseline, and his body temperature was kept at 33°C. Urine output was constant at 40 ml/hr. He came off CPB requiring two inotropes and two vasoconstrictors. Even so, his systolic blood pressure was low, and his pulse pressure narrows. He was then started on methylene blue which improved his MAP. On arrival to the intensive care unit (ICU), he immediately required continuous veno-veno haemodialysis (CVVHD) and developed acute liver failure. At 16 hours, he showed a clinically fair neurological recovery. Forty-eight hours post-surgery, he suffered multiorgan failure and developed an intractable arrhythmia and died. The unusual components were as follows: he was normally responsive to phenylephrine during CPB; despite normal rSO_2_ and a clinically neurological recovery, he suffered multiorgan failure; and his serial high-sensitivity (HS) troponin I levels never fell below 500,000 pg/ml (normal <14 pg/ml).

## Introduction

Vasoplegia syndrome (VS) can lead to vasoplegic shock. It is a type of vasodilatory shock that usually occurs during or early after cardiopulmonary bypass (CPB), but delayed onset has been reported [1]. When VS develops into shock, it is classified as distributive shock. The commonest cause of VP is sepsis. However, of all causes of shock, VS accounts for only 5% of cases. The second commonest cause of VS is the type described here and typically follows CPB during cardiac surgery. Approximately 5% of patients undergoing cardiac surgery may experience degrees of VS. When shock develops, it has a mortality of up to 25% [2,3]. The usual clinical presentation is during prolonged CPB with an increasing tolerance to vasoconstrictors over time. The main clinical feature is low blood pressure while maintaining a normal or raised cardiac output (CO). Once started, the ability to maintain a desired mean arterial blood pressure (MAP) is lost despite the use of a combination of vasoconstrictors. Flow is maintained, but perfusion pressure to vital organs is reduced. The anaesthetist looks at physiological responses that indicate adequate perfusion despite low pressure. During CPB, urine output is always considered an important indicator of renal perfusion. Patients with renal dysfunction preoperatively will be more susceptible to ischaemic damage if perfusion pressure is low. In our patient, in addition to urine output, we measured regional brain cerebral oxygen saturation (rSO_2_) to indicate cerebral perfusion and oxygenation. It is often considered that the brain can act as an index organ whereby if the brain is perfused so other organs will be too. The purpose of this report is to highlight not only the rapid onset of VS but that the brain did not function as the index organ for this patient. Once the patient is off CPB, the additional indicators of the adequacy of CO are temperature and end-tidal carbon dioxide (EtCO_2_). 

Outcomes associated with vasoplegic syndrome include multiorgan failure, prolonged length of stay in the ICU and mortality [4]. Vasoplegia syndrome becomes vasoplegic shock when there is catecholamine-resistant hypotension with normal or augmented CO, with a cardiac index (CI) greater than 2.2 l/kg/m^2^ and systemic vascular resistance (SVR) less than 800 dynes·s/cm^5^ with signs of major organ dysfunction and hypoperfusion manifested by raised lactate. 

Many drugs (e.g., preoperative angiotensin-converting enzyme inhibitors) have been cited as risk factors, along with multiple comorbidities and low ejection fraction. However, prolonged aortic cross-clamp and CPB times are the most consistent risk factors.

The pathophysiology of VS is complex involving diminished nitric oxide levels, abnormal movement of potassium across the cell membrane, low levels of endogenous vasopressin and diminished smooth muscle receptor response to catecholamines. It may be that VS is a spectrum with some patients developing shock within a time frame that other patients can tolerate [4].

## Case presentation

A 37-year-old, 112-kg male patient presented with progressively worsening shortness of breath and lower limb oedema. He had been diagnosed with rheumatic heart disease one year previously. Transoesophageal echocardiography (TOE) reported severe mitral regurgitation with an eccentric jet with mild mitral stenosis and mild to moderate aortic regurgitation. Both valves showed features of rheumatic heart disease. He declined surgery at the time due to his own concerns about his renal transplant, and he felt his symptoms were mild. His symptoms now caused him to reconsider. He was getting increasing shortness of breath and ankle oedema. Preoperatively, he was fully mobile, but exercise tolerance was reduced. He had no symptoms of infection or chest pain. His chest was clear, and on the day of surgery, his temperature was 36.1°C and blood pressure (BP) 130/77. His laboratory results for the pre- and postoperative period are shown in Table 1.

**Table 1:** Laboratory Data Pre-and Postoperatively Swan-Ganz catheter data postoperatively. ICU: intensive care unit, POD: postoperative day, eGFR: estimated glomerular filtration rate, CVVHD: continuous veno-veno haemodialysis, ALT: alanine aminotransferase, AST: aspartate aminotransferase, LDH: lactate dehydrogenase, WBC: white blood cells, HS: high sensitivity, SVRI: systemic vascular resistance index, PVRI: pulmonary vascular resistance index, PAWP: pulmonary artery wedge pressure, MAP: mean arterial pressure, bpm: beats per minute.

Parameter with normal values and units	Preoperative values	ICU values admission	ICU values POD 1	ICU values POD 2
eGFR (>60 l/min/1.73 m^2^)	45	26	27 (on CVVHD)	53 (on CVVHD)
Creatinine (65-112 µmol/l)	164	257	255	144
ALT (7-44 U/l)	12	-	602	4,569
AST (5-34 IU/l)	14	-	>913	>913
Bilirubin (3.4-22 mmol/l)	9	-	17.1	41.4
LDH (100-217U/l)	288	-	>3,325	-
WBC (4-11 x10^9^/l)	14.1	35.7	46.6	46.5
Lactate (0.7-2 mmol/l)	-	11.57	14.54	13.26
Procalcitonin (0.25 µg/l)	-	-	93.27	209.73
HS troponin I (1.9-34.2 pg/ml)	-	>500,000	>500,000	>500,000
Cardiac index (3.0-5.0 l/min/m^2^)	-	4.37	2.1	-
SVRI (1200-2,000 dynes·s/cm^5^/m^2^)	-	914	1,895	-
PVRI (225-315 dynes·s/cm^5^/m^2^)	-	201	332	-
PAWP (mmHg)	-	26	22	-
MAP (mmHg)	80	65	70	-
Heart rate (bpm)	85	126	79	-

The patient had a renal transplant in 2017. His renal function was impaired, and he required regular diuretics and immunosuppressive medications. Preoperative medications include prednisolone 5 mg once daily (od), mycophenolate 1,500 mg od, furosemide 80 mg od, metoprolol 25 mg od, amlodipine 10 mg od and hydralazine 75 mg od.

Preoperative TOE findings confirmed a rheumatic mitral valve with mean gradient of 4.6 mmHg, significant mitral regurgitation with an eccentric jet and a rheumatic aortic valve with mild to moderate aortic stenosis and regurgitation. Ejection fraction was 50%-55%. He had normal coronary arteries. The patient was scheduled for mechanical mitral and aortic valve replacement. 

**Figure 1:**
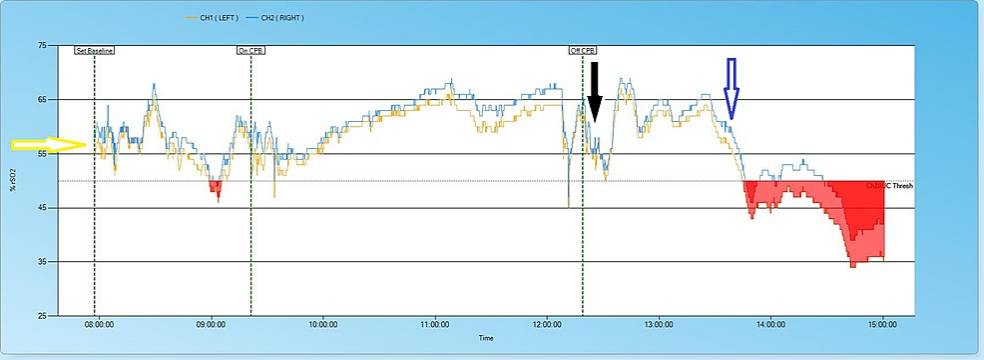
Cerebral Oximetry Values From Induction of Anaesthesia to Transfer to Intensive Care Yellow arrow indicates baseline cerebral oximetry (rSO_2_) indicating normal baseline of 57%. Black arrow indicates weaning off cardiopulmonary bypass. Blue arrow indicates start of methylene blue infusion which creates artificially low readings below threshold shown as red shaded area. CH: channel, CPB: cardiopulmonary bypass, rSO2: regional cerebral oxygen saturation.

Surgery was commenced under general anaesthesia including supplementary hydrocortisone 100 mg. Standard monitoring of invasive arterial and central venous blood pressure, nasopharyngeal temperature and urine output was applied including rSO_2_ with baseline values taken awake breathing room air. The TOE probe was inserted after induction of anaesthesia. Baseline rSO_2_ was 57% with one dip pre-CPB during cannulation. During CPB, rSO_2_ values are maintained above baseline. At the first attempt to wean CPB, the rSO_2_ dropped and full-flow CPB was resumed. On the second weaning attempt, the rSO_2_ dipped briefly and is then well above baseline until the administration of methylene blue (1 mg/kg) which rapidly creates false low readings. Values for the whole case are shown in Figure 1.

**Figure 2:**
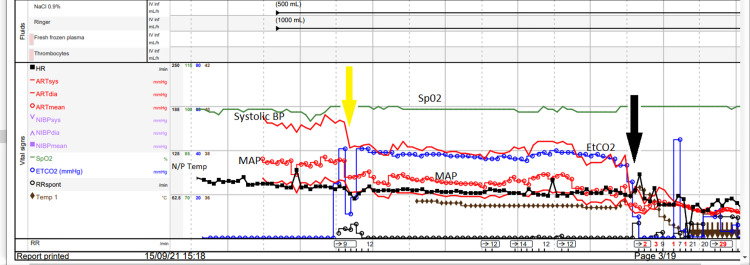
Intraoperative Monitoring From Induction of Anaesthesia to Start of Cardiopulmonary Bypass Yellow arrow indicates induction of anaesthesia. Black arrow indicates start of cardiopulmonary bypass. HR: heart rate, RR: respiratory rate, ARTsys: systolic arterial pressure, NIBP: non-invasive blood pressure (not recorded), RRspont: spontaneous respiratory rate (not recorded), SpO2: peripheral saturation, systolic BP: systolic blood pressure, MAP: mean arterial pressure, EtCO2: end-tidal carbon dioxide, N/P temp: nasopharyngeal temperature.

**Figure 3:**
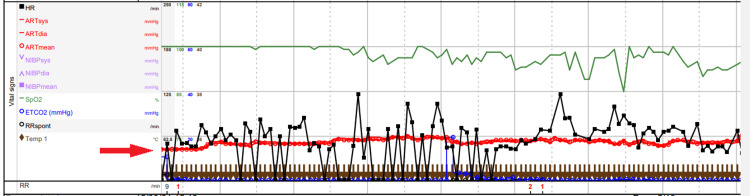
Intraoperative Monitoring During Cardiopulmonary Bypass Red arrow indicates mean arterial pressure during cardiopulmonary bypass maintained between 60 and 70 mmHg. The grey line just above the red circles is the 70 mmHg pressure line. HR: heart rate, RR: respiratory rate, ARTsys: systolic arterial pressure, ARTdia: diastolic arterial pressure, SpO2: peripheral saturation, EtCO2: end-tidal carbon dioxide, NIBP: non-invasive blood pressure, RRspont: spontaneous respiratory rate (not recorded).

**Figure 4:**
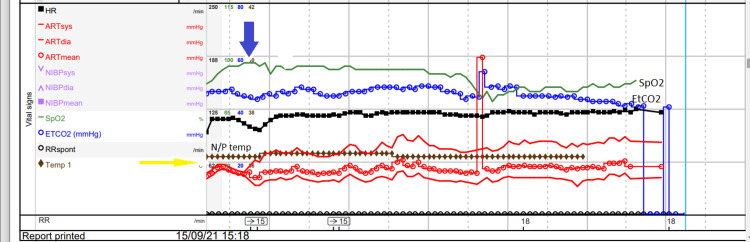
Intraoperative Monitoring for the Period From Weaning Off Cardiopulmonary Bypass to Transfer to Intensive Care Yellow arrow indicates the 70 mmHg line on the arterial pressure scale. Blue arrow indicates start of methylene blue infusion. HR: heart rate, RR: respiratory rate, ARTsys: systolic arterial pressure, ARTdia: diastolic arterial pressure, SpO_2_: peripheral saturation, EtCO_2_: end-tidal carbon dioxide, N/P temp: nasopharyngeal temperature maintained above 36.0^o^C, NIBP: non-invasive blood pressure, RRspont: spontaneous respiratory rate (not recorded).

The monitoring of other intraoperative data throughout the case is shown in Figures 2-4 which divide the surgical periods. Figure 2 shows the period from induction of anaesthesia to initiation of CPB. This was uneventful with normal MAP at 70 mmHg and peripheral saturation (SpO_2_) >97% and the patient requiring no vasoactive drug support.

Figure 3 shows the period on CPB. MAP is maintained close to 60 mmHg throughout. Urine output during CPB was 40 ml/hr, and ultrafiltration was used directly from the CPB circuit. His surgery during CPB involved replacement of the mitral valve with a St Jude 29 mitral valve and replacement of the aortic valve with a St Jude 21 aortic valve. The total time for the CPB was 204 minutes. During CPB, only regular boluses of phenylephrine (0.1 mg) were required. Anaesthesia was maintained with sevoflurane, cisatracurium and fentanyl.

At the first attempt to come off CPB, the left atrium became severely dilated. The mechanical mitral valve was opening, but the left ventricle was not ejecting. Figure 4 shows second weaning off CPB with the patient on milrinone 0.43 µg/kg/min, noradrenaline 0.05 µg/kg/min, adrenaline 0.05 µg/kg/min and dual chamber pacing. At this time, despite a MAP of 50 mmHg and a narrow pulse pressure, the surrogate markers of cardiac output, namely, rSO_2_, temperature and end-tidal carbon dioxide (EtCO_2_), indicated an adequate CO. As the left ventricle (LV) was now contracting better on the TOE, due to the low MAP, vasopressin 0.04 µg/kg/min was added. As the inotrope doses increased and there was little response to vasopressin, we realized this was a vasoplegia syndrome and started methylene blue. The administration of methylene blue (1 mg/kg) rapidly increased the MAP and the pulse pressure. Both the rSO_2_ and the SpO_2_ values are affected by the methylene blue. Figure 4 clearly shows how the MAP and systolic blood pressure improved with methylene blue and allowed us to partially close the chest and move to ICU.

In ICU, he was started on continuous veno-veno haemodialysis (CVVHD) as he was now acidotic and anuric. As his MAP improved and his CO was sufficient, the milrinone was stopped. He received nitric oxide 20 parts per million (ppm) to off-load his right ventricle and maintain oxygenation with fractional inspired oxygen (FiO_2_) 0.4. By postoperative day (POD) 1, the other vasoactive drugs were all reduced compared with the doses required in ICU the previous evening, but he required CVVHD for persistent renal and liver failure. Also on the POD 1, it was possible to reduce his sedation and assess his neurological function; he responded normally. As we planned to close the chest the next day, he was kept sedated and ventilated.

Table 1 shows the results indicating multiorgan failure. The patient continued on prophylactic antibiotics but clinically was not considered to be septic. The rise in procalcitonin was attributed to surgical trauma and multiorgan failure. There were no positive cultures. Also shown are the haemodynamic values from the Swan-Ganz catheter. On POD 2, an attempt was made to close the chest during which the patient became unstable and developed ventricular tachycardia progressing to ventricular fibrillation. With cardioversion, he resumed a sinus tachycardia, but acidosis and organ dysfunction rapidly progressed, and he died a few hours later in multiorgan failure.

## Discussion

Vasoplegia syndrome can occur in any condition in which there is a profound inflammatory response that is rapidly unresponsive to vasopressors. In ICU, VS is commonly associated with sepsis, burns and trauma. Our case occurred as a result of cardiac surgery in which prolonged CPB time is a known contributing factor. There is no precise length of time, but with longer CPB, the incidence of VS increases. In cardiac surgery, it is defined as mean arterial pressure <65 mmHg without hypovolaemia that is unresponsive to vasoconstrictor therapy. The definition requires the exclusion of low cardiac output states caused by ventricular failure, use of inodilators, tamponade, evidence of inadequate tissue perfusion and the absence of sepsis [5]. Whether VS is caused by sepsis or cardiac surgery, lactate level is elevated in the shock state [6]. The clinical dilemma is the balance between the adverse effects of high-dose vasoconstrictors and the need for adequate perfusion pressure which will vary according to the premorbid major organ function. For example, the perfusion pressure for the brain, kidney, liver and gastrointestinal tract may all differ as might the susceptibility to excessive use of vasoconstrictors. In this case, the patient had two major contributing factors for developing VS, a long CPB time and preexisting renal failure. A study of dialysis patients requiring CPB for cardiac surgery demonstrated an incidence of VS of 30% and, amongst this group, a mortality of VS of 50% [7]. Although our patient was not on dialysis preoperatively, he had had a previous renal transplant which was not functioning normally. Therefore, this may have been another risk factor.

Recognizing VS requires prompt action and the use of drugs that are not regularly administered in routine cardiac anaesthesia. Treatment is based on the past two decades of cases and studies looking at many vasoconstrictors alone or in combination [8,9]. Therapies also include non-catecholamine drugs such as methylene blue [10]. Liver toxicity has been reported after using methylene blue but at much higher doses than typically used for VS.

At the time of planning to come off CPB, there was no warning of vasoconstrictor resistance. During the 204 minutes on CPB only, intermittent boluses (0.1 mg) of phenylephrine had been required. However, as the pump flow reduced, the left atrium specifically dilated and the heart was not ejecting. After inserting a dual chamber pacemaker and starting inotropes and vasoconstrictors, the patient came off CPB. Despite low MAP, his surrogate markers for CO, namely, rSO_2_ readings above baseline, temperature maintained above 36°C and the EtCO_2_ >5 kPa or 38 mmHg in the post-CPB period, indicated a good CO. These surrogate indicators of CO supported the VS diagnosis. This had not been the case on CPB when his CO was maintained by the pump and his BP maintained with increments of phenylephrine. Further confirmation of VS was the response to methylene blue as shown by an increase in MAP and pulse pressure. A few hours later in ICU, the Swan-Ganz catheter data showed a high CO.

One unexpected finding was the first and serial high-sensitivity (HS) troponin I values that were all >500,000 pg/ml indicating very significant myocardial damage. In the setting of cardiac surgery, such HS troponin I values would indicate a poorly performing heart and the use of mechanical support to augment the patient’s CO to avoid multiorgan failure. Mechanical support was discussed but ruled out due to the patient’s own high CO. The second point of discussion was that throughout the operation, the rSO_2_ values were consistently above baseline even during the period of low MAP. It is often considered in cardiac surgery that if the cerebral perfusion is maintained, as indicated by rSO_2_ values, in the normal range, then the other major organs of the body will be perfused. The concept being that the brain is the index organ [11]. Clinically, the brain was fully functional when the sedation was decreased and the patient responded to verbal commands 16 hours postoperatively. Despite this, during the next 24 hours, he progressed into renal and liver failure and acidosis, leading to intractable dysrhythmias and death 48 hours post-surgery. Although the brain may act as an index organ for many patients, this was not the case in our patient.

What could we have done differently? Although he was perfusing his brain as measured clinically and by rSO2, he was not perfusing his other major organs. We focused on renal function and started CVVHD, but perhaps mechanical support of the left ventricle would have been a better option. Also, the very high HS troponin I values mean that mechanical support would have decreased his myocardial oxygen requirement rather than increasing oxygen consumption with inotropes. This could have reduced the propensity to dysrhythmia. Our treatment of adrenaline, noradrenaline and vasopressin plus hydrocortisone followed by methylene blue is conventional. Less established adjunctive therapies include vitamin C, hydroxocobalamin, thiamine and calcium along with alternations to the CPB circuit [12].

## Conclusions

We present a case of vasoplegia syndrome which develops into vasoplegic shock in a young patient with two risk factors, prolonged CPB and a previous renal transplant which was failing. Post-CPB, he rapidly went into shock despite prompt conventional treatment for VS and died in multiorgan failure within 48 hours of surgery. This was despite monitoring brain oxygenation, which had been adequate throughout, thereby cerebral oxygenation did not provide protection as an index organ. We therefore wish to highlight with this case, the speed with which VS can develop and the importance of being up-to-date with a range of drugs not normally used in cardiac anaesthesia along with newer adjunctive therapies for VS and a surgical plan for mechanical support if significant myocardial muscle loss occurs. Also to be aware that even if the rSO_2_ is normal and cardiac output is maintained, an adequate perfusion pressure is required to prevent multiorgan failure and a high mortality.
